# Botanical Description of British Plants

**Published:** 1805-12

**Authors:** 


### *Botanical Description of British Plants.*

[ Continued from p. 169—174. ]

8. Erica. *E. vulgaris.*

*Ang.* Common heath. Ling. Grig, *in Shropshire*; Hather, *in Scotland.*

*Gen. Desç.* Cal. four-leaved. Bloss. four-cleft. Filaments fixed to the receptacle. Anthers, cloven and perforated at top. Caps. four celled, four-valved, many seeded.

*Spec.*

---

*Spec. Dec. Anthers with two tooth-serrated awns at the base; leaves opposite;* arrow-shaped. Cal. has close to its base four or five circular, concave, coloured leaves, fringed with soft hairs, and outside of these, two or three partly resembling these, and partly the leaves of the cup. *Proper cup* coloured, resembling exactly the *blossom*, which is pale rose colour, sometimes white, not distended. *Heaths, Woods.* Bl. June, August.

*Use.* This plant is but little regarded in happier climates, but, in the bleak and barren Highlands of Scotland, it is made subservient to a great variety of purposes. In the Isle of Islay, ale is frequently made by brewing, one part malt and two parts of the young tops of heath: to which they sometimes add hops. Boethius relates that this liquor was much used by the Picts. The poorer inhabitants make walls for their cottages, with alternate layers of heath and a kind of mortar made of black earth and straw, the woody roots of the heath being placed in the centre, the tops externally and internally. They make their beds of it by placing the roots downwards; and the tops only being uppermost, they are sufficiently soft to sleep upon. Cottages are thatched with it also.—*Pennant.* Woollen cloth boiled in alum water, and afterwards in a strong decoction of the tops of heath, comes out a fine orange colour. The stalks and tops will tan leather. In England, besoms are made of it, and faggots to burn in ovens or to fill up drains that are to be covered over. Bees extract a great deal of honey from the flowers, but where heath abounds the honey has a reddish cast. Sheep and goats will sometimes eat the tender shoots, but are not fond of them.—*Withering.*

9. Populus. *P. alba.*

*Ang.* White Poplar. Abele-tree.

*Gen. Desc.* Fl. M. and F. in catkins on distinct plants. Calyx, scales ragged. Bloss. turban-shaped, mouth entire, slanting. Fem. summit four-cleft, caps. two-celled, seeds many, downy.

*Spec. Desc. Leaves* nearly triangular, toothed, angular, blackish green, white thick cotton underneath; *leaf-stalks* flatted, grooved on each side. *Tree* very tall. *Hedges, woods, and near brooks.* Bl. March.

*Use.* The wood is soft, white and stringy, and makes good wainscoting, being little subject to swell or shrink. Floors, laths, packing boxes, and turner's ware are made of it. It loves low situations, and flourishes best in clay: it grows quick and bears cropping, but is unfavourable to pasturage—

pasturage.—*Withering.* The wood has the advantage of not being liable to take fire and never blazing.—*D. B.* Horses, sheep, and goats eat it: cows do not.

10. Populus. *P. tremula.*

*Ang.* Asp. Aspen. Aspen-tree. Trembling Poplar.

*Gen. Desc.* *As above.*

*Spec. Desc.* *Leaves* circular, toothed, angular, smooth on both sides, rolled inwards, with two glands running one into another on the inner side above the base. *Leaf-stalks* flatted at the end. *Moist woods, boggy ground.* Bl. March, April.

*Use.* The wood is extremely light, white, and smooth, woolly, soft, and durable in the air. The bark of the young trees is made into torches. The bark of this tree is the principal food of beavers. It will grow in every situation and in all soils, but worst in clay: it impoverishes the land; its leaves destroy the grass, and the numerous shoots of its roots spread so near the surface of the earth, that nothing else can grow there. It is easily transplanted. Sheep and goats eat it: horses and swine refuse it.—*Withering.*

11. Populus. *P. nigra.*

*Ang.* Black Poplar.

*Gen. Desc.* *As above.*

*Spec. Desc.* *Leaves* trowel-shaped, tapering to a point, serrated, smooth on both sides, without glands at the base, but the serratures glandular at the inside. *Stamens* sixteen. *Leaf-stalks* yellowish. *Near rivers, wet shady places.* Bl. March.

*Use.* The bark, being light like cork, is used by fishermen for supporting their nets. The wood is not apt to splinter. It loves a moist black soil, grows rapidly, and bears cropping. Horses, cows, sheep, and goats eat it.—*Withering.*

12. Daphne. *D. Mezereum-Daphnoides. Thymelæa. Chamelæa germanica.*

Ang. Mezereon. Spurge Olive. Spurge Flax. Dwarf Bay.

*Gen. Desc.* Cal. generally none. Bloss. one-petal, regular, four-cleft, funnel-shaped: drupa like a berry, one-celled, superior.

*Spec. Desc.* *Leaves* spear-shaped, deciduous, from the terminating bud. *Flowers* from the lateral buds, sitting on the stem, mostly three together, opening early in spring, very thick set, fine red: *berry* globular red. *Woods near Andover.* Bl. February, March.

*Use.*

*Use.* This plant is extremely acrid, especially when fresh, and if retained in the mouth, occasions great and long continued heat and inflammation, particularly of the throat and fauces: the berries have the same effect, and, when swallowed, form a powerful corrosive poison not only to man, but to dogs, wolves, foxes, &c. The bark and berries, in different forms, especially prepared in an ointment, have long been applied to ill-conditioned sores and ulcers: in France the former is strongly recommended as an application to the skin, which, under the following management, (unknown in this country) produces a continued serous discharge, without blistering, useful in many chronic diseases, and answering the purpose of what is called a perpetual blister, with less pain and inconvenience, viz. A square piece of the recent bark, about an inch long and three-quarters of an inch broad, macerated in a little vinegar, is applied to the skin, over which is bound a leaf of ivy or plantane: this application is at first renewed night and morning, till it cauterizes the part and brings on a serous discharge, after which a renewal of the bark once in twenty-four hours is sufficient to continue the issue for any length of time. By means of suitable plasters, it might probably be applied behind the ears, to relieve the eyes; and on a larger scale, prove a useful practice in sundry diseases. It must be observed, however, that it sometimes produces cutaneous eruptions, which Bergius attributes to the absorption of the acrid particles of the bark. In this country, the Mezereon is principally employed in *syphilitic* complaints. See *Monro's Ess. & Obs. Phys. & Lit. iii. p.* 402. Dr. Russel has established the utility of the *cortex mezerei* in *venereal nodes.* "A decoction made of two drachms of the bark of the root, boiled in three pints of water till one pint is wasted, and this quantity drank daily, is found efficacious in resolving venereal nodes and other indurations of the periosteum." See *Med. Obs. & Inq. iii. p.* 189. Dr. R. never found the decoction increase any of the natural evacuations. Dr. Home, as it is observed by Dr. Cullen, has not only found this decoction cure scirrhous tumours, which remain after the lues venerea and after the use of mercury, but that it healed also some scirrhous tumours from other causes; and he has employed it in several cutaneous affections, sometimes with success. See *M. M. ii. p.* 215.—*Woodville.* The considerable and long continued heat and irritation that it produces in the throat, when chewed, induced Dr. Withering to give it in a case of difficulty ficulty in swallowing, seemingly occasioned by a paralytic affection: the patient chewed a thin slice of the root as often as she could bear to do it; and in about two months she recovered her power of swallowing. This woman bore, with great resolution, the disagreeable irritation and the ulcerations that its acrimony occasioned in her mouth, but she had been reduced to skin and bone, and for three years before had suffered extremely from hunger, without being able to satisfy her appetite, for she could not swallow solids at all, and liquids but very imperfectly: the complaint came on after lying-in.—*Withering.* The whole plant is very corrosive, so much so that six berries will kill a wolf. A woman gave twelve grains of the berries to her daughter, who had a quartan ague; she vomited blood and died immediately.—*Linn.* As the acrimony of these berries is not perceived upon tasting them, the ignorant and unwary are the more easily betrayed to swallow them. The root is an *antiscorbutic,* and has been ordered with sweetening woods. *H. B.*

13. DAPHNE. *D. laureola.*

*Ang.* Spurge Laurel. Laurel mezereon.

*Gen. Desc.* *As above.*

*Spec. Desc.* *Bunches* of five flowers, axillary, nodding, clustered into an umbel. *Leaves* spear-shaped, smooth. *Floral-leaves* concave, alternate, without any flowers from their base. *Bloss.* yellow green. *Woods, hedges.* Bl. March, April.

*Use.* Very happy effects have been experienced from this plant in rheumatic fevers: it operates as a brisk and rather severe purgative. It is an efficacious medicine in worm cases; and upon many accounts deserves to be better known to Physicians; but in unskilful hands, it would be dangerous, as it is possessed of very considerable acrimony. The whole plant has the same qualities, but the bark of the root is the strongest. Dr. Alston fixes the outside dose at ten grains.—*Withering.*

OCTANDRIA. DIGYNIA.

14. CORYLUS. *C. avellana.*

*Ang.* Common hazel-nut tree.

*Gen. Desc.* M. and Fem. fl. distinct, on the same plant. Bloss. 0. M. cal. one leaf, three-cleft, resembling a scale, with one flower. F. cal. two-leaved, ragged. Nut egg-shaped.

*Spec. Desc.* *Stipulæ* spear-shaped, *twigs* hairy. *Styles* vivid crimson: *catkins* in pairs, green; when out of flower brown. *Scale,* middle segment pointed at the end. *Leaves* oval, serrated, wrinkled. *Woods, hedges.* Bl. March, April.

*Use.* In countries where yeast is scarce, it is a common practice practice to take twigs of hazel, and twisting them together so as to be full of chinks, to steep them in the ale during its fermentation; they are then hung up to dry, and at the next brewing they are put into the wort instead of yeast. In Italy the chips are frequently put into turbid wine for the purpose of clearing it, which is effected in twenty-four hours. The wood is much used for fishing-rods, walking sticks, crates, hoops, &c. and the shoots for sprin-gles to fasten down thatch. Where beautiful woods is required for inlaying or staining, the roots of the hazel are preferred. Painters and engravers prepare from this wood, charcoal for sketching their designs: they cleave the timber to about the thickness of a finger, put the pieces into a pot filled with sand, cover it over with clay, expose it in a potter's oven, or any other sufficient heat, and when cooled the sticks are found converted into charcoal. The nuts are agreeable to most people: an expressed oil is obtained from them, for the use of painters. Squirrels mostly live upon them. Hazel is frequently planted in hedges and coppices, to make charcoal for forges; and being cut down in equal portions, in the rotation of sixteen years, they produce a considerable regular revenue. Horses and goats eat the leaves; sheep and swine refuse them.—*Withering*.

OCTANDRIA. TRIGYNIA.

15. POLYGONUM. *P. hydropiper*.

*Ang*. Water-pepper. Arse-mart. Lakeweed. Biting Snakeweed.

*Gen Desc.* Cal. 0. Bloss. resembling a cup, five-div. seed one, angular, generally naked.

*Spec. Desc. Stamens six; caps. one-celled. Pistils* cloven; *Stipulæ* somewhat fringed; *leaves* waved, spear-shaped, not spotted, very thin, smooth, entire, with bristles laid to the edge, scarcely perceptible. *Spikes*, long, slender, drooping. *Flowers* green, red toward the end. The whole plant sprinkled with minute glandular dots, even with the surface. *Watery places*. BL. July, Sept.

*Use*. The whole plant has an acrid burning taste. It cures little *apthous ulcers* in the mouth. The ashes of this plant, mixed with soft soap, is a nostrum in a few hands, for dissolving the *stone* in the bladder; but it may be questioned whether it has any advantage over other semi-caustic preparations of the vegetable alkali. Its acrimony rises on distillation, and the distilled water drank to the amount of two or three pints daily, has been found effectual in some *nephritic* cases. It dyes wool yellow. All cattle refuse it.—*Withering*.

( To be continued. )

